# Nickel Ion Inhibits Nuclear Factor-Kappa B Activity in Human Oral Squamous Cell Carcinoma

**DOI:** 10.1371/journal.pone.0068257

**Published:** 2013-07-03

**Authors:** Takashi Shionome, Shigeki Endo, Daisuke Omagari, Masatake Asano, Hitoshi Toyoma, Tomohiko Ishigami, Kazuo Komiyama

**Affiliations:** 1 Department of Partial Denture Prosthodontics, Nihon University School of Dentistry, Tokyo, Japan; 2 Department of Pathology, Nihon University School of Dentistry, Tokyo, Japan; Johns Hopkins University, United States of America

## Abstract

**Background:**

The spontaneous IL-8 secretion observed in OSCC is partially dependent on the disregulated activity of transcription factor NF-κB. Nickel compounds are well established human carcinogens, however, little is known about the influence of nickel on the spontaneous secretion of IL-8 in oral squamous cell carcinoma (OSCC) cells. The aim of the present study was to investigate whether Ni^2+^ ions can influence on IL-8 secretion by OSCC.

**Methods and Results:**

The IL-8 secretion was measured by ELISA. The expression of IL-8 mRNA was examined by real-time PCR. The NF-κB activity was measured by luciferase assay. The phosphorylation status and nuclear localization of NF-κB subunits were examined by Western blotting or Transfactor kit and immunofluorescence staining, respectively. The interaction of NF-κB p50 subunit and Ni^2+^ ions was examined by Ni^2+^-column pull down assay. The site-directed mutagenesis was used to generate a series of p50 mutants. Scratch motility assay was used to monitor the cell mobility. Our results demonstrated that, on the contrary to our expectations, Ni^2+^ ions inhibited the spontaneous secretion of IL-8. As IL-8 reduction was observed in a transcriptional level, we performed the luciferase assay and the data indicated that Ni^2+^ ions reduced the NF-κB activity. Measurement of p50 subunit in the nucleus and the immunofluorescence staining revealed that the inhibitory effect of Ni^2+^ ions was attributed to the prevention of p50 subunit accumulation to the nucleus. By Ni^2+^-column pull down assay, Ni^2+^ ions were shown to interact directly with His cluster in the N-terminus of p50 subunit. The inhibitory effect of Ni^2+^ ions was reverted in the transfectant expressing the His cluster-deleted p50 mutant. Moreover, Ni^2+^ ions inhibited the OSCC mobility in a dose dependent fashion.

**Conclusions:**

Taken together, inhibition of NF-κB activity by Ni^2+^ ion might be a novel therapeutic strategy for the treatment of oral cancer.

## Introduction

Nickel compounds are well established human carcinogens, with occupationally exposed nickel refinery workers and miners having an increased incidence of lung and nasal cancer [1] [2]. Nickel compounds may be water soluble or water insoluble. All nickel compounds are carcinogenic, but water-insoluble compounds, such as nickel subsulfide, are more potent than water-soluble compounds [3]. Nickel compounds cause DNA hypermethylation, histone deacetylation and chromatin condensation, which may play an important role in their carcinogenicity by decreasing the transcription of tumor suppressor and senescence genes [4]. Ni^2+^-containing alloys are commonly used in dental applications [5]. Ni^2+^ ions released from dental materials may cause not only cancer but also inflammatory diseases such as oral lichen planus, which is similar to a hypersensitivity reaction [6]. Patch testing of dental materials revealed that Ni^2+^ ions are the most common allergen [7]. The surface of the oral cavity is covered by oral epithelial cells (OECs). Histologically, OECs are stratified squamous epithelial cells sharing common properties with skin-derived epithelial cells [8]. OECs play a pivotal role in protecting the underlying connective tissue from invading pathogens. The most frequently occurring malignant tumor in the oral cavity is oral squamous cell carcinoma (OSCC) [9], which is one of the six most common cancers. Carcinogenesis is generally considered to be a multistep accumulation of genetic alterations. One of the most important mechanisms is usually the loss of tumor suppressor functions, such as p16 inactivation and mutation in the p53 gene. Nickel, however, being highly carcinogenic, is only a weak mutagen and is not expected to contribute directly to the mutation process [4]. However, some of the aberrant gene and protein expression observed in OSCC is dependent on the disregulated activity of transcription factor NF-κB [9]. The NF-κB transcription factors are assembled by the dimerization of five family members: p50 (NFKB1), p52 (NFKB2), p65, also known as RelA (RELA), c-Rel (REL), and RelB (RELB) [10] which, upon activation, translocate to the nucleus where they participate in the expression of genes involved in inflammatory and immune responses, as well as in cell proliferation and survival [11]. NF-κB protein levels increase gradually from premalignant lesions to invasive cancer, indicating the important role of NF-κB at the early stages of carcinogenesis [12] [13] [14] [15]. Interfering with NF-κB activity leads to a remarkable reduction in the number of cytokines and chemokines, including IL-2, IL-6 and IL-8 [16]. One of the most relevant factors for the growth of OSCC is IL-8, which induces angiogenesis [17]. The aim of the present study was to investigate whether OSCC can respond to Ni^2+^ ions and augment the secretion of IL-8. Contrary to our expectations, Ni^2+^ ions inhibited the secretion of IL-8 in OSCC. The molecular mechanisms underlying the inhibitory effect of Ni^2+^ ions was investigated. The results indicate that Ni^2+^ ions can inhibit nuclear translocation of the NF-κB p50 subunit. By examining the possible direct interaction between Ni^2+^ ions and the p50 subunit, we confirm that Ni^2+^ ions can bind directly with the histidine cluster in the N-terminal part of the p50 subunit and prevent the nuclear translocation. We propose a novel mechanism by which Ni^2+^ ions can regulate the activity of NF-κB in OSCC.

## Materials and Methods

### Reagents

Nickel chloride and L-1–4′-tosylamino-phenylethyl-chloromethyl ketone (TPCK) were purchased from Sigma (St. Louis, MO). Isohelenin was purchased from Calbiochem (Tokyo, Japan). Antibodies (Abs) against human TLR4, p65 and p50 were purchased from Santa Cruz (Santa Cruz, CA). Anti-phospho-p65 Ab was purchased from Cell Signaling Technology (Tokyo, Japan). Anti-GAPDH Ab was purchased from Chemicon (Tokyo, Japan). HRP-conjugated goat anti-rabbit IgG (H+L) Ab and HRP-conjugated goat anti-mouse IgG (H+L) Ab were purchased from Jackson ImmunoResearch (West Grove, PA, USA) and FITC-conjugated goat anti-rabbit IgG (H+L) was purchased from Zymed (South San Francisco, CA).

### Cell Culture and Ni^2+^ Ions Stimulation

Human OSCC cell lines HSC2, HSC3 and Ca9–22 cells were obtained from Health Science Research Resources Bank (Osaka, Japan) and maintained by RPMI1640 supplemented with 10% fetal calf serum (FCS), 50 µg/ml streptomycin and 50 U/ml penicillin (10% FCS-RPMI) [18], [19]. Human umbilical vein endothelial cells (HUVECs) were maintained with Endothelial Cell Basal Medium-2 (Lonza, Walkersville, MD, USA). To test Ni^2+^ ions stimulation, 2×10^5^ cells were plated in a 24-well dish on the day before experiment. The cells were washed with 10% FCS-RPMI twice and further cultured in the presence or absence of various concentrations of Ni^2+^ ions for 24 h. For Ab blocking experiments, the cells were pre-incubated with 2 µg of anti-TLR4 Ab or class-matched Ab for 1 h. After washing, the cells were stimulated with Ni^2+^ ions for 24 h. To test NF-κB inhibition, cells were pre-incubated with varying concentrations of either TPCK or isohelenin for 30 min or 1 h, respectively. Stimulation with Ni^2+^ ions was performed as described above.

### IL-8 Measurement

After Ni^2+^ ion stimulation, the culture supernatants were harvested. Samples were cleared by centrifugation and subjected to enzyme-linked immunosorbent assay (ELISA). IL-8 concentration was measured by DuoSet ELISA Development System (R&D Systems, Tokyo, Japan). The absorbance was measured on a microplate reader model 3550 (Bio Rad, Tokyo, Japan).

### Reverse Transcriptase-polymerase Chain Reaction (RT-PCR)

Total RNA was purified using RNeasy mini kit (QIAGEN, Tokyo, Japan). cDNA was synthesized with Superscript III reverse transcriptase (Invitrogen, San Diego, CA) and subjected to RT-PCR as described previously [20]. Real-time PCR was performed using LightCycler nano (Roche, Tokyo, Japan) with SYBR green (TaKaRa, Tokyo, Japan). The primers used in this study were as follows: TLR4 5′-TGGATACGTTTCCTTATAAG-3′ (forward), 5′-GAAATGGAGGCACCCCTT-C-3′ (reverse); MD2, 5′-ATGTTACCATTTCTGTTTTTTTC-3′ (forward), 5′-GATTAAACTTAATCCAACCAC-3′ (reverse); β-actin 5′-GGAGCAAGTATCTTGATCTTC-3′ (forward), 5′-CCTTCCTGCGCATGGAGTCCTG-3′ (reverse); IL-8 5′-ATGACTTCCAAGCTGGCC-3′ (forward), 5′-CTTCTCCACAACCCTCTGC-3′ (reverse).

### Immunofluoresence Staining

HSC3 cells were plated on cover slips at a density of 5 ×10^5^/35-mm dish. The cells were washed with 10% FCS-RPMI twice and further cultured in the presence or absence of 1 mM Ni^2+^ ions for 1 h. At the end of stimulation, the cells were washed with PBS twice and fixed in 2% paraformaldehyde-PBS (Polysciences, Warrington, PA) for 10 min. The cells were permeabilized with 1% TritonX 100-PBS for 10 min and washed three times with PBS. Non-specific staining was blocked by incubating the cells with 1% bovine serum albumin (BSA)-PBS for 20 min. The cells were incubated with rabbit anti-human p50 Ab for 1 h (×100 dilution with 1% BSA-PBS). After incubation, the cells were washed with PBS three times and further incubated with FITC-conjugated goat anti-rabbit IgG (H+L) Ab (×100 dilution with 1% BSA-PBS). After washing, nuclear staining was performed with monomeric cyanine nucleic acid stain (Invitrogen,) and mounted on glass slide with Aqua-Polymount (Polysciences). The images were captured with a LSM510 confocal laser microscope (Carl Zeiss, Heidelberg, Germany).

### SiRNA Experiment

The siRNAs were purchased from Invitrogen. The cells were plated on the day before transfection at a density of 2×10^5^/35-mm dish. The cells were washed three times with OPTI-MEM (Invitrogen) and then transfected using the Lipofectamine RNAi/MAX transfection method (Invitrogen) according to the manufacturer’s instruction. Briefly, 100 pmol of RNAi duplex was diluted in 250 µl of OPTI-MEM, and separately 6 µl of Lipofectamine RNAi/MAX was diluted in 250 µl of OPTI-MEM, each in an eppendorf tube. These two solutions were mixed and incubated for 20 min at room temperature. The transfection mixture was applied to HSC3 cells and incubated for 3 h at 37°C in a 5% CO_2_ incubator. After incubation, the cells were washed with 10% FCS-RPMI three times and further cultured for 3 h. The cells were washed and cultured with or without Ni^2+^ ions for 24 h. The culture supernatants and total RNA were harvested and subjected to ELISA and real-time PCR, respectively.

### Luciferase Assay

HSC3 cells were plated in 48-well culture plates at a density of 1×10^5^ cells/well. The cells were washed twice with OPTI-MEM and transfected with 1 µg of reporter plasmids (pNF-κB-Luc, Stratagene, Tokyo, Japan) using the Lipofectamin transfection method (Invitrogen). After 3 h of transfection, the cells were washed with 10% FCS-RPMI and further cultured for 3 h. The cells were washed and either left unstimulated or stimulated with 1 mM Ni^2+^ ions for 1 h. After stimulation, the cells were lysed with 1× passive lysis buffer (Promega, Tokyo, Japan) and cell lysates were collected. Transfection efficiency was normalized to renilla luciferase activity by co-transfection with the pRL/CMV vector (Promega). Both firefly and renilla luciferase activities were determined using the Dual-Luciferase Reporter Assay System (Promega). Luminescence was measured on a Lumat LB9507 luminometer (Berthold, Bad Wildbad, Germany).

### Western Blotting

HSC3 cells were stimulated with or without 1 mM Ni^2+^ ions for 30, 60, 120 and 180 min. After stimulation, the cells were washed with ice cold PBS twice and lysed with 500 µl of cell lysis buffer (50 mM Tris-HCl, pH 7.5, 150 mM NaCl and 0.5% TritonX-100). The protein concentration was measured using BioRad protein assay kit (BioRad) and 100 µg of total protein was subjected to 10% SDS-PAGE. Western blotting was performed as described previously [20]. The primary Abs against total p65, phosphorylated p65 and GAPDH were diluted to ×1,000 with 1% BSA-PBST (0.1% tween-20/PBS). The secondary goat anti-mouse IgG (H+L) and goat anti-rabbit IgG (H+L) were diluted to ×10,000 with 1% BSA-PBST. The protein expression level of p65 and phosphorylated p65 after treatment with or without NF-κB inhibitor, TPCK or isohelenin, was also examined by Western blotting.

### Measurement of NF-κB Subunits

For the detection of NF-κB subunits localized to the nucleus, HSC3 cells were stimulated with or without 1 mM Ni^2+^ ions for the indicated times. The nuclear extracts were prepared with TransFactor cell extraction kit (Clontech, CA, USA). The protein concentrations were measured with BioRad protein assay kit (BioRad) and 100 µg of total protein were subjected to Transfactor kit (Clontech).

### DNA Construction and Stable Transfection

The full length complementary DNA of NF-κB p50 subunit was obtained from Kazusa DNA Research Institute (Chiba, Japan) and the insert was excised and subcloned to expression vector pcDNA3.1(+) (Invitrogen). This clone was designated as pcDNA-p50 wild type (WT). The N− and C-terminal half of p50 was amplified with PCR using the following primers p50 N-primer 5′-ATGGCAGAAGATGATCCATA-3′ (forward), 5′-GAGCCGCACCACGCTGAGGT-3′ (reverse); p50 C-primer, 5′-ATGTTTACAGCTTTTCTTCC-3′ (forward), 5′-CCCAGCATTAGATTTAGTAG-3′ (reverse), and subcloned to pcDNA3.1(+) vector (pcDNA-p50 N and pcDNA-p50 C), respectively. The mutant lacking His residues at positions 108, 110 and 112 was constructed using Quickchange site-directed mutagenesis kit (Invitrogen). For stable transfection, pcDNA-p50 WT, pcDNA-p50 N and pcDNA-p50 C plasmids were linearised by PvuI digestion. Transfection was performed for 4 h with lipofectamine reagent as described previously [21]. The transfection solution was replaced with 10% FCS-RPMI and further cultured for 18 h. Cells were then split 1∶5 in media containing 0.4 µg/ml geneticine (Sigma). The medium was changed every 3 days. Colonies were picked up after 10 days of culture and expanded. For transient transfection, cells were infected with recombinant vaccinia virus prior to transfection as described previously [22].

### Ni^2+^-column Precipitation

After transient transfection, the cells were lysed with cell lysis buffer and the protein concentration was measured with Bio Rad protein assay kit (Bio Rad). One hundred µg of total protein were mixed with either 10 µl of Ni^2+^-column (GE healthcare, Tokyo, Japan) or protein G-sepharose column (GE Healthcare) and rotated for 18 h at 4°C. The samples were washed five times with cell lysis buffer and subjected to 10% SDS-PAGE. The separated protein was transferred to Immobilone membrane (Millipore, Tokyo, Japan). Western blotting was performed with rabbit anti-p50 Ab (×100) (Santa Cruz) followed by HRP-conjugated goat anti-rabbit IgG (H+L) Ab.

### Scratch Motility Assay

For the scratch motility assay, 2.5×10^5^/cells were seeded on 12-well plates. A plastic pipette tip was used to scratch the cell monolayer to create a cleared area, and the cells were washed with fresh medium to remove loose cells. Immediately following scratching (0 h) and after incubation of cells at 37°C for 24 h, cells were stained with crystal violet solution (2% crystal violet/0.8% oxalic acid, diammonium salt in methanol) for 1 min. The cells were washed with PBS and phase-contrast images of the cell motility process were photographed digitally with Nikon Eclipse Ti microscope with DS-Fi1 camera (Nikon, Tokyo, Japan). The distance of the clear areas were measured on the images, set at 100% for 0 h, and the mean percentage of the total distances of the wound areas was calculated. The migration of cells was monitored microscopically.

### Statistical Analysis

Results are presented as means±SD of at least three independent experiments. Statistical differences were assessed using the Student’s t-test, Welch’s test or Steel-Dwass test. Significant differences (P<0.05) are indicated.

## Results

### Ni^2+^ Ions Inhibit the Spontaneous Secretion of IL-8 in OSCC Cell Lines

**Figure 1:**
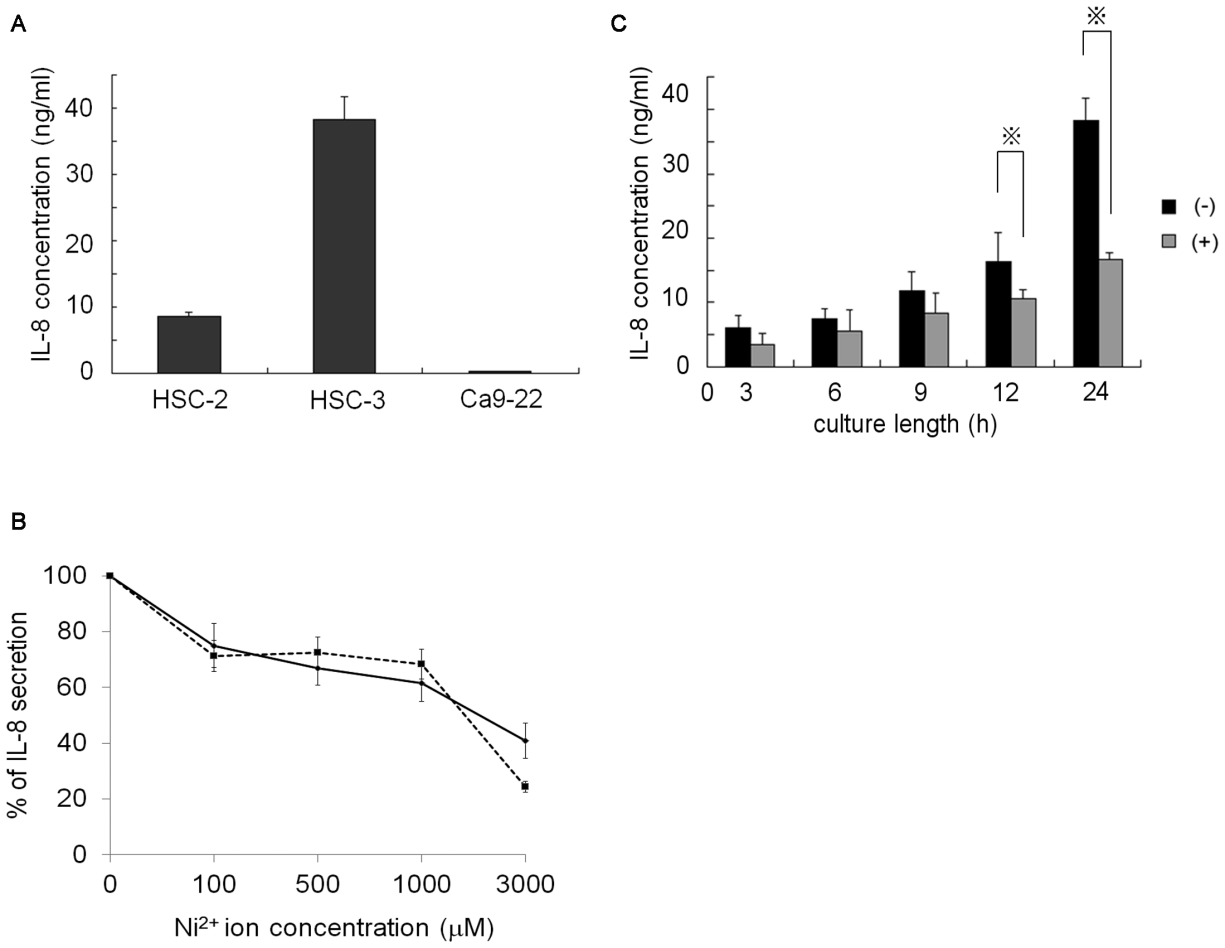
Spontaneous secretion of IL-8 in OSCC cell lines. Cells (2×10^5^) were plated in a 24-well dish on the day before the experiment. (A) The cells were washed with 10% FCS-RPMI twice and further cultured for 24 h. The culture supernatants were harvested and IL-8 concentration was measured by ELISA. (B) HSC3 (solid line) and HSC2 cells (dotted line) were stimulated with various concentrations of Ni^2+^ ions for 24 h. The amount of IL-8 secreted in the absence of Ni^2+^ ions was set as 100%. (C) HSC3 cells were cultured with 1 mM Ni^2+^ ions for the indicated times and IL-8 concentration was measured. Data are representative of five separate experiments (mean ±SD). *p<0.05.

To examine endogenous IL-8 production in OSCC, culture supernatants were harvested and IL-8 concentration was measured by ELISA. As shown in Figure 1A, the highest secretion of IL-8 was observed in HSC3 cells. The concentration of IL-8 in the HSC3 cell supernatant was 38 ng/ml, while that of HSC2 cells was 8.5 ng/ml. In Ca9–22 cells, IL-8 secretion was observed, but the concentration was lower than 250 pg/ml. These results confirm spontaneous secretion of IL-8 by OSCC cell lines. Next, the effect of Ni^2+^ ions on the secretion of IL-8 was examined. HSC3 cells were chosen for these experiments because of their high levels of IL-8 secretion. HSC3 cells were cultured in the presence or absence of varying concentrations of Ni^2+^ ions for 24 h and the concentration of IL-8 was measured. Surprisingly, secretion of IL-8 decreased in a Ni^2+^ ion concentration-dependent manner, reaching an IC_50_ of 3 mM (Figure 1B). Fresh medium to which Ni^2+^ ions were added did not show any absorbance, indicating the specific measurement of IL-8. Ni^2+^ ion-mediated IL-8 inhibition was also observed with HSC2 (Figure 1B). Since 10% cell death was observed in samples containing 3 mM of Ni^2+^ ions, we chose to add 1 mM to the samples for the following experiments. To investigate the time course of Ni^2+^ ion inhibition, HSC3 cells were cultured for the times indicated in Figure 1C. The inhibitory effect of Ni^2+^ ions was not apparent within 9 h of incubation. After 12 h of incubation, the inhibitory effect gradually appeared, and at 24 h IL-8 secretion was reduced by 50%.

### Ni^2+^ Ions Inhibit NF-κB Activity

**Figure 2:**
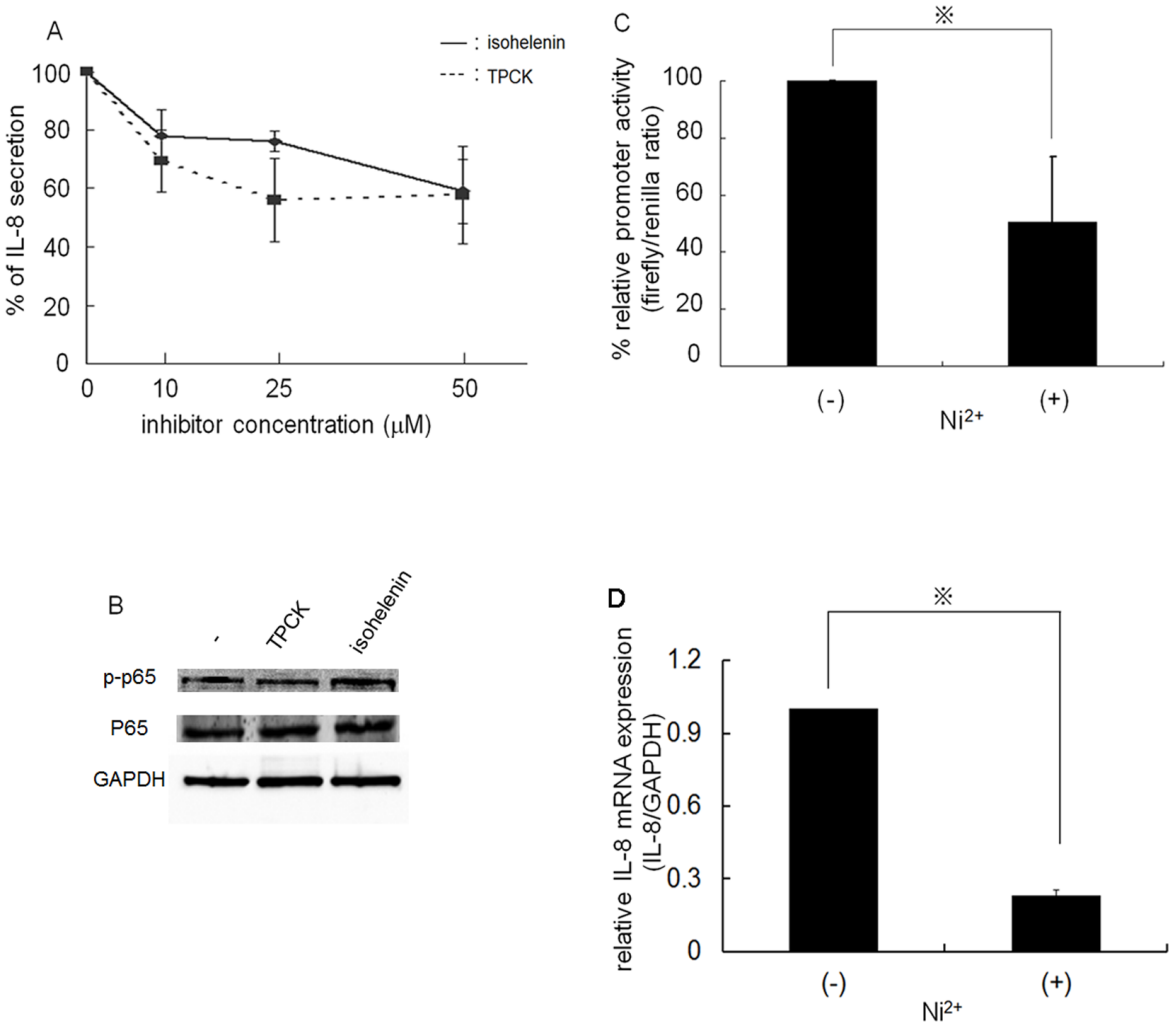
NF-κB-dependent secretion of IL-8. (A) HSC3 cells were exposed to different concentrations of the NF-κB-specific inhibitors isohelenin (solid line) and TPCK (dashed line). After 24 h, secreted IL-8 concentration was measured. Data from at least three separate experiments are shown (mean±SD). (B) Total cell lysates were collected after treatment of HSC3 cells with or without TPCK or isohelenin and subjected to Western blotting. The membranes were probed with anti-phospho-p65 Ab (top panel), anti-p65 Ab (middle panel) or anti-GAPDH Ab (lower panel). Representative data for three separate experiments are shown. (C) HSC3 cells were transfected with pNF-κB-Luc plasmid (firefly) and pRL-CMV plasmid (renilla). After transfection, cells were stimulated with (+) or without (–) 1 mM Ni^2+^ ions for 1 h. Luciferase activity was measured and the firefly/renilla ratio was calculated. The ratio in the absence of Ni^2+^ ion stimulation was set as 100%. Data are means±SD of three independent experiments. *p<0.05. (D) HSC3 cells were stimulated as in (B). After stimulation, the expression of IL-8 mRNA was measured with real-time PCR. The IL-8/GAPDH ratio in the absence of Ni^2+^ ions was set as 1. *p<0.05.

Spontaneous IL-8 secretion in several cancer cell lines is reported to be due to aberrant NF-κB activity [9]. To confirm NF-κB-dependent IL-8 secretion, HSC3 cells were pre-incubated with various concentrations of the NF-κB-specific inhibitors TPCK and isohelenin. After treatment, the cells were further incubated in the presence or absence of 1 mM Ni^2+^ ions and IL-8 concentration was measured. In HSC3 cells, IL-8 secretion decreased upon exposure to each inhibitor in a concentration-dependent fashion (Figure 2A). Both inhibitors reached IC_50_ values of 50 µM. These results indicate that spontaneous IL-8 secretion was at least partially due to autonomous activation of NF-κB in OSCC. The protein expression level of p65 and phosphorylated p65 after pre-incubation with TPCK or isohelenin was examined by Western blotting. Neither p65 nor phosphorylated p65 level was drastically changed by the treatment (Figure 2B). As demonstrated above, spontaneous IL-8 secretion was inhibited by Ni^2+^ ions. We speculated that Ni^2+^ ion-mediated IL-8 inhibition could be due to inactivation of NF-κB activity. To test this possibility, we performed a luciferase assay. HSC3 cells were transfected with pNF-κB-Luc plasmid and incubated with or without 1 mM Ni^2+^ ions. Promoter activity was expressed relative to the internal control. In the absence of Ni^2+^ ions, high NF-κB activity was observed. When the relative firefly/renilla ratio was set as 100%, this value was reduced to 60% in the presence of 1 mM Ni^2+^ ions (Figure 2C). These results suggest that Ni^2+^ ions have a direct inhibitory effect on NF-κB activity. If this is the case, Ni^2+^ ions should reduce IL-8 mRNA levels. Based on this assumption, HSC3 cells were incubated with or without Ni^2+^ ions and the expression of IL-8 mRNA was examined using real-time PCR. As expected, Ni^2+^ ions inhibited the expression of IL-8 mRNA to 25% of levels recorded in the absence of Ni^2+^ ions (Figure 2D). These results clearly demonstrate that spontaneous IL-8 secretion in OSCC can be attributed to aberrant NF-κB activity and that Ni^2+^ ions exert an inhibitory effect through inactivation of NF-κB.

### The Inhibitory Effect of Ni^2+^ Ions is Independent of TLR4

**Figure 3:**
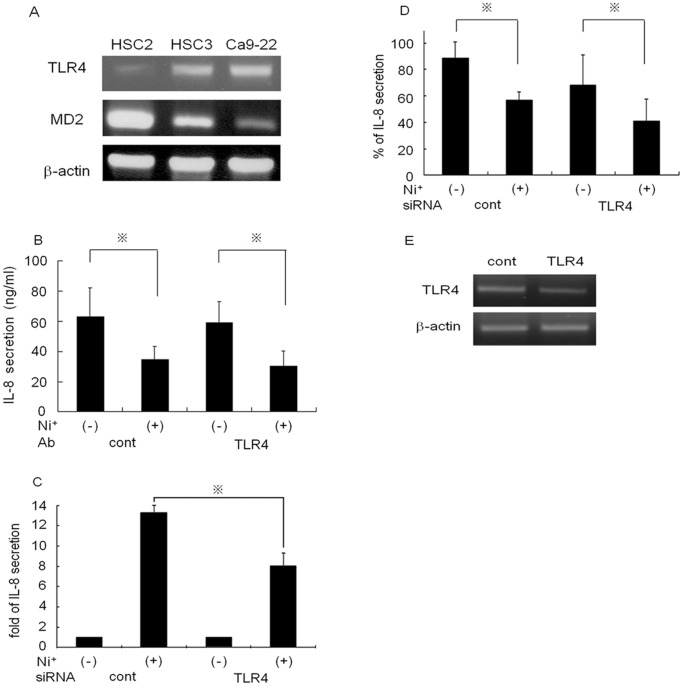
Ni^2+^ ions exerts an inhibitory effect through a TLR4-independent pathway. (A) Expression of TLR4 and MD2 mRNA in HSC2, HSC3 and Ca9–22 cells was examined using RT-PCR. Expression levels varied significantly among the cells. (B) HSC3 cells were pre-incubated with 2 µg anti-TLR4 Ab or class-matched control Ab for 2 h. After pre-incubation, the cells were stimulated with or without 1 mM Ni^2+^ ions for 24 h. The culture supernatants were harvested and subjected to IL-8 ELISA. Data for the three separate experiments are shown (mean±SD). *p<0.05. HUVECs (C) and HSC3 cells (D) were transfected with TLR4 siRNA or control siRNA. The transfectants were cultured in the presence or absence of 1 mM Ni^2+^ ions for 24 h. IL-8 concentration was measured by ELISA. The IL-8 concentration of control siRNA-transfected HUVECs cultured without Ni^2+^ was set as 1 and the fold increase was shown (C). The IL-8 concentration of non-transfected HSC3 cells cultured without Ni^2+^ was set as 100% (D). Data are means±SD of three independent experiments. *p<0.05. (E) HSC3 cells were transfected with TLR4 siRNA or control siRNA. After transfection, TLR4 expression was examined by RT-PCR. Representative values for three independent experiments are shown.

Recently, Ni^2+^ ions have been shown to interact with the extracellular part of TLR4 and can evoke contact hypersensitivity reactions in humans [23] . We speculated that Ni^2+^ ions inhibit IL-8 secretion through TLR4. To explore this possibility, we first examined the expression of TLR4 and its adaptor MD2 in OSCCs using RT-PCR. Both TLR4 and MD2 expression was observed in all cell lines (Figure 3A), but at significantly varying levels of expression. TLR4 expression was most prominent in HSC3 and Ca9–22 cells, and lowest in HSC2 cells. By contrast, the highest level of MD2 expression was observed in HSC2 cells and to a lesser extent in HSC3 cells. MD2 expression was much lower in Ca9–22 cells. Based on these results, we next examined whether TLR4 contributed to Ni^2+^ ion-mediated inhibition of IL-8 secretion. HSC3 cells were pre-incubated with anti-TLR4-specific Ab or a class-matched control Ab for 2 h. The cells were washed and further cultured for 24 h in the presence or absence of 1 mM Ni^2+^ ions. The supernatants were collected and IL-8 concentratins were measured by ELISA. Neither control- nor anti-TLR4 Ab pre-treatment affected spontaneous IL-8 seccretion. IL-8 concentrations decreased from 61 to 36 ng/ml in control Ab-treated cells and from 59 to 31 ng/ml in anti-TLR4 Ab-treated cells, respectively (Figure 3B). These results indicated that the addition of anti-TLR4 Ab did not influence the secretion of IL-8. The contribution of TLR4 to Ni^2+^ ion-mediated IL-8 reduction was further examined using siRNA. Since Ni^2+^ ions induce IL-8 secretion in HUVECs [23], HUVECs were used as positive controls. The control- or TLR4-siRNA transfected cells were incubated with or without Ni^2+^ ions and IL-8 was measured by ELISA. In HUVECs, Ni^2+^ ions augmented the secretion of IL-8 in a control siRNA transfection (13-fold induction, IL-8 concentration without Ni^2+^ ion stimualation was set as 1) (Figure 3C). TLR4 siRNA transfection reduced the secretion of IL-8 to 8-fold. By contrast, in HSC3 cells, both the control- and the TLR4-siRNA transfection by itself reduced the spontaneous secretion of IL-8 to 90% and 70% respectively of that in siRNA non-transfected cells. When the cells were incubated with 1 mM Ni^2+^ ions, IL-8 secretion was further reduced in both the control (60%) and the TLR4 siRNA transfectants (55%) (Figure 3D). Reduction of TLR4 mRNA was confirmed by RT-PCR, and it was shown that control siRNA had no effect on the TLR4 mRNA level (Figure 3E). Taken together, these results indicate that the inhibitory effect of Ni^2+^ ions is a TLR4-independent process.

### Ni^2+^ Ions Affect Subcellular Localization of the NF-κB p50 Subunit

**Figure 4:**
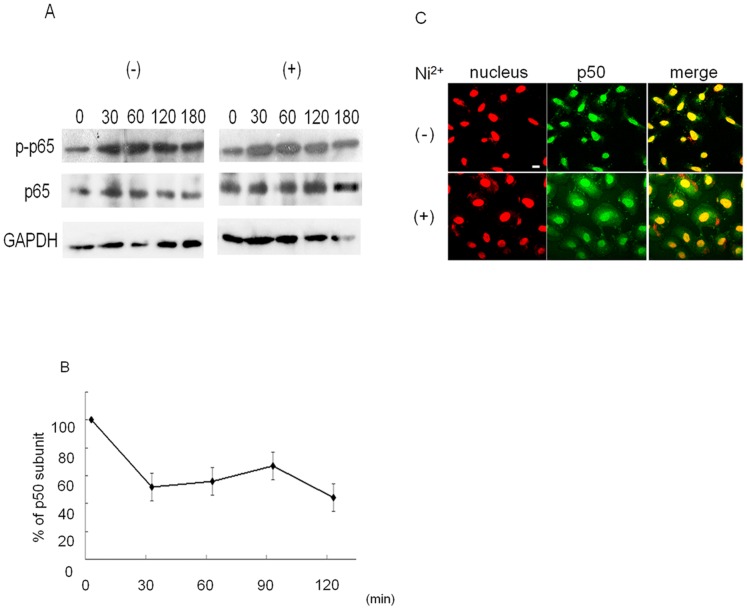
Ni^2+^ ions inhibit the nuclear translocation of the NF-κB p50 subunit. (A) HSC3 cells were stimulated with 1 mM Ni^2+^ ions (or with medium as a control) for the indicated times. Cell lysates were prepared and subjected to Western blotting. The membranes were probed with anti-phospho-p65 Ab (top panel), anti-p65 Ab (middle panel) or anti-GAPDH Ab (lower panel), respectively. Representative data for three separate experiments are shown. (B) HSC3 cells were stimulated with 1 mM Ni^2+^ ions for the times indicated. The nuclear extracts were harvested and subjected to a Transfactor assay to measure p50. Data are means±SD of three independent experiments. (C) HSC3 cells were stimulated with 1 mM Ni^2+^ ions for 1 h. After stimulation, cells were immediately transferred to ice, washed with ice-cold PBS and fixed. The cells were subjected to immunofluorescence cell staining with anti-human p50 Ab followed by FITC-conjugated goat anti-rabbit IgG Ab. Green, p50; red, nuclei with monomeric cyanine nucleic acid stain. Bar, 10 µm.

NF-κB activity is regulated by several different steps [11]. To examine which step is affected by Ni^2+^ ions, we first examined the phosphorylation status of NF-κB. Since IL-8 expression is regulated by the classical NF-κB pathway [24], p65 phosphorylation was examined. HSC3 cells were cultured with or without Ni^2+^ ions for the indicated times (Figure 4A). After stimulation with Ni^2+^ ions, cell lysates were prepared and subjected to Western blotting. Throughout the stimulation, the amount of total p65 and GAPDH was constant (Figure 4A, middle and lower panel) and no drastic changes were observed in the phosphorylation level of p65 regardless of the presence of Ni^2+^ ions (Figure 4A, upper panel).

Next, the nuclear translocation of the NF-κB subunits p65 and p50 was examined. HSC3 cells were stimulated as described above and nuclear extracts were prepared. The amount of each subunit was measured using a TransFactor kit (Clontech). Although the amount of p65 in the nucleus was unchanged (data not shown), stimulation of the cells with Ni^2+^ ions significantly reduced the amount of p50 (Figure 4B). The level of p50 subunit in the nucleus decreased to 50% after 30 min of stimulation and was maintained even after 2 h of stimulation. These results suggest that Ni^2+^ ions could affect the nuclear translocation of p50. To confirm these observations, immunofluorescence staining was performed using anti-p50 Ab. HSC3 cells were cultured in the presence or absence of Ni^2+^ ions for 1 h. The cells were immediately placed on ice to stop translocation and were stained with anti-p50 Ab. In the absence of Ni^2+^ ions, most p50 localized to the nucleus (Figure 4C, upper panel). By contrast, when the cells were cultured with Ni^2+^ ions, a significant amount of p50 localized to the cytoplasm (Figure 4C, lower panel). These results indicate that Ni^2+^ ions affect the subcellular localization of p50 subunit.

### Direct Binding of Ni^2+^ Ions Bind Directly to the p50 Subunit

**Figure 5:**
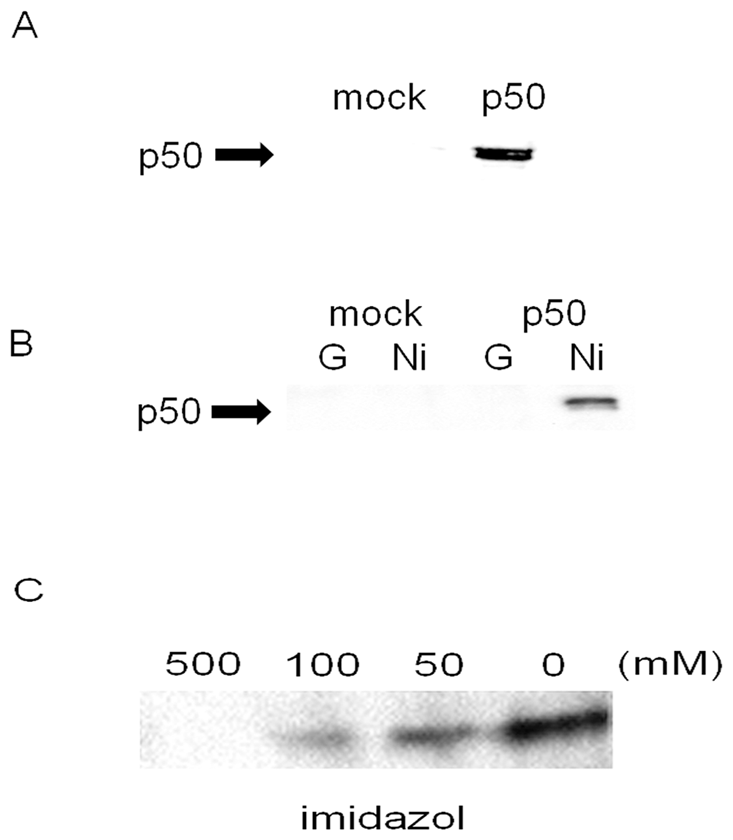
Ni^2+^ ions bind directly to the p50 subunit of NF-κB. (A) HSC3 cells were transfected with p50 expression vector (pcDNA-p50 WT). Cell lysates were harvested and subjected to Western blotting. (B) The cell lysates of pcDNA-p50 WT or mock-transfected cells were collected and incubated with either Ni^2+^-column (Ni) or protein G-sepharose beads (G) for 1 h. The samples were washed with ice-cold cell lysis buffer five times and subjected to Western blotting. (C) The cell lysates from p50-transfected HSC3 cells were incubated with Ni-beads for 1 h in the presence or absence of graded concentrations of imidazol. The samples were subjected to Western blotting.

The inhibitory effect of Ni^2+^ ions on IL-8 secretion could be mediated by direct binding of Ni^2+^ ions to p50. To examine this possibility, p50 was force expressed in HSC3 cells by recombinant vaccinia virus-mediated transfection. The high level expression of p50 was confirmed by Western blotting (Figure 5A). Cell lysates were prepared from both p50 transfected or mock transfected cells and incubated with either a Ni^2+^-column or with protein G-sepharose beads. After incubation, the columns were washed and subjected to Western blotting to detect p50 interaction. As shown in Figure 5B, p50 was only detected when p50 transfected cell lysate was incubated with the Ni^2+^-column but not with the protein G-sepharose beads. As the Ni^2+^ binds to its target through a histidine residue (His), a binding assay was performed in the presence of graded concentrations of imidazol. The amount of p50 decreased according to the concentration of imidazol and the binding was totally lost in the presence of 500 mM of imidazol (Figure 5C). These data indicate that p50 can interact with Ni^2+^ ions and this interaction is mediated through the His residue.

### The His Cluster is Indispensable for p50 and Ni^2+^-column Binding

**Figure 6:**
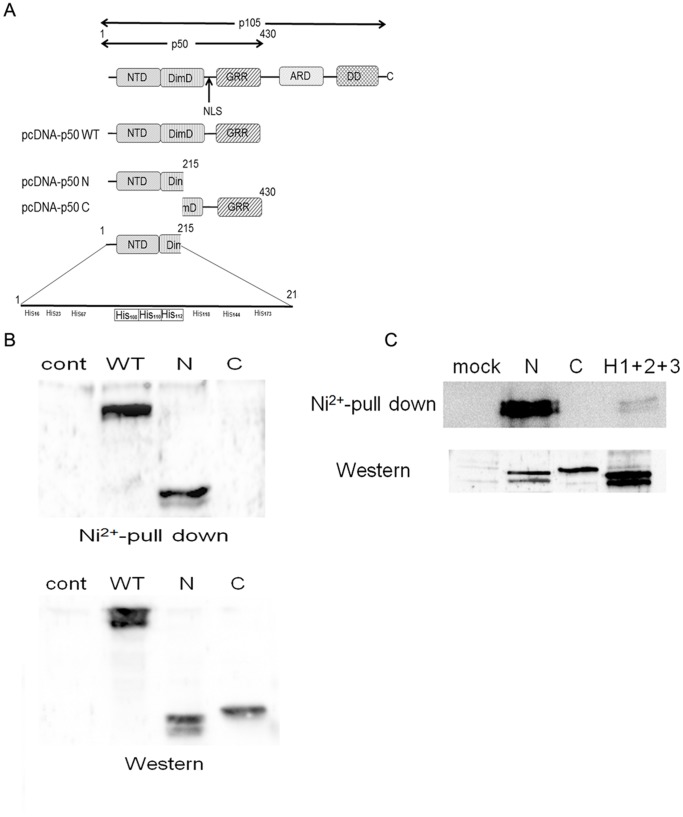
Ni^2+^ ions bind to the N-terminal part of the p50 subunit. (A) Schematic illustration of the structure of the p105 molecule. NTD; N-terminal domain, DimD; dimerization domain, NLS; nuclear localizing signal, GRR; glycine-rich region, ARD; ankyrin repeat domain, DD; death domain. The lower panel shows the His clusters at position 108, 110 and 112. (B) HSC3 cells were transfected with mock, pcDNA-p50 WT, pcDNA-p50 N or pcDNA-p50 C plasmids. The cell lysates were harvested and subjected to Ni**^2+^**-column precipitation (upper) or Western blotting (lower). (C) The His cluster was deleted and transfected to HSC3 cells. The cell lysate was harvested and subjected to a Ni^2+^-column precipitation assay followed by Western blotting.

To further explore the binding site, we constructed an expression plasmid containing full length (pcDNA-p50 WT), N-terminal parts (pcDNA-p50 N) or C-terminal parts (pcDNA-p50 C) of the p50 subunit (Figure 6A). The structure of the p50 subunit is illustrated in Figure 6A. Each fragment was subcloned to the expression vector pcDNA3.1. The plasmids were transfected and a Ni^2+^-column binding assay was performed. As shown in Figure 6B (upper panel), the lysates from pcDNA-p50 WT and pcDNA-p50 N transfectants showed clear p50 bands, but those from pcDNA-p50 C did not. The expression of each protein was confirmed with the cell lysates obtained from each transfectant (Figure 6B, lower panel), indicating that the N-terminal part of the p50 molecule can bind to the Ni^2+^-column.

As the N-terminal half of p50 contains nine His residues (Figure 6A), we further examined which His residue contributes to binding with the Ni^2+^-column. The position of each His residue is shown in Figure 6A. A cluster of His residues was found in positions 108, 110 and 112. We attempted to delete these three His residues by site-directed mutagenesis (H1+H2+H3) and performed a Ni^2+^-column binding assay. Western blotting demonstrated successful expression of all mutants (Figure 6C, lower panel). Consistent with above experiments, clear binding was detected with pcDNA-p50 N, but not with pcDNA-p50 C (Fig. 6C, upper panel). However, the binding was significantly reduced in a mutant lacking all three His residues (H1+H2+H3) (Figure 6C, upper panel). These results indicate the indispensable role of the His cluster for direct binding of Ni^2+^ ions to p50.

### Overexpression of the p50 N-terminal Reverses the Inhibitory Effect of Ni^2+^ Ions

**Figure 7:**
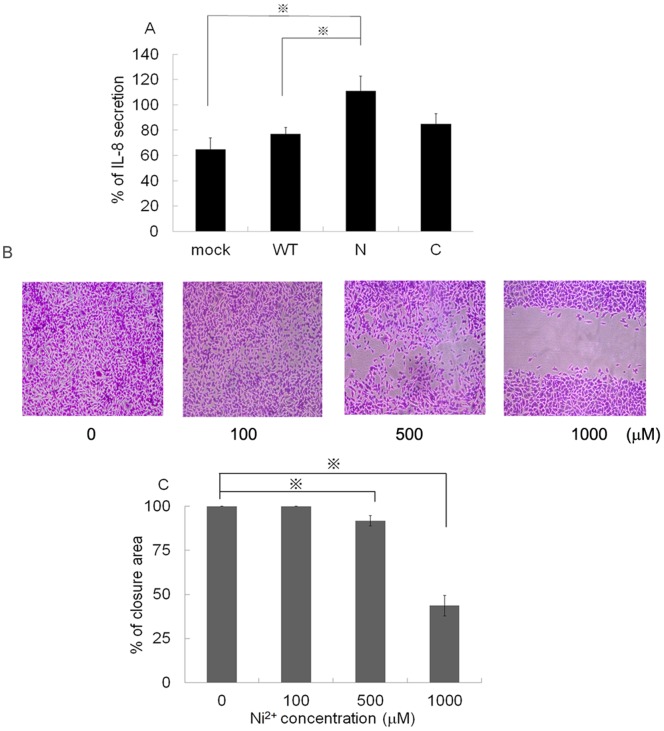
Overexpression of the N-terminal part of p50 blocks the inhibitory effect of Ni^2+^ ions. (A) Stable transfectants expressing mock, WT, N- or C-terminal parts of p50 were established. Each transfectant was cultured in the presence of 1 mM Ni^2+^ ions for 24 h. After stimulation, the culture supernatants were harvested and subjected to IL-8 ELISA. The concentration of IL-8 in mock transfectant cultured in the absence of Ni^2+^ ions was set as 100%. The relative IL-8 secretion is shown. Data are means±SD of three independent experiments. *p<0.05. (B) Migration of HSC3 cells was determined with a scratch motility assay. At 18 h after scratching the cells, phase-contrast images (5×fields) of the scratch motility process were obtained. Representative images are shown. (C) Each scratched area was measured on the images, set at 100% for 0 h, and the mean percentage of the total closure of the scratched area was calculated.

If the inhibitory effect of Ni^2+^ ions is mediated by direct binding to the His cluster in the N-terminal part of p50, overexpression of the p50 N-terminal could prevent the inhibitory effect of Ni^2+^ ions. To examine this possibility, we established stable transfectants expressing WT, N- or C-terminal parts of the p50 subunit, and mock transfectant. The transfectants were cultured in the presence or absence of 1 mM Ni^2+^ ions and the IL-8 concentrations were measured. The IL-8 concentration of the culture supernatant obtained from the mock transfectant was set as 100%. When the mock transfectant was cultured in the presence of Ni^2^ ions, IL-8 secretion of 64% was observed (Figure 7A). Although the pcDNA-p50 WT and pDNA-p50 C transfectants showed slight increases in IL-8 concentration to 76% and 85% respectively, these were not statistically significant (p = 0.30 for pcDNA-p50 WT and p = 0.42 for pDNA-p50 C). In contrast, the inhibitory effect of Ni^2+^ ions was markedly impaired with the pcDNA-p50 N transfectant (p<0.032). IL-8 concentration increased significantly to 111% of the mock transfectant level (Figure 7A). These results indicate that the N-terminal part of the p50 subunit can reverse the inhibitory effect of Ni^2+^ ions, and its overexpression abrogates Ni^2+^ ion-mediated IL-8 reduction in HSC3 cells.

### Ni^2+^ Ions Inhibit the Migration Activity of OSCC

NF-κB activity correlates with the metastatic potential of cancer cells [10] . Highly metastatic cancer cells have higher NF-κB activity. If Ni^2+^ ions reduce NF-κB activity, they could inhibit the motility of OSCC. To explore these possibilities, we performed a scratch motility assay. Although 1 mM Ni^2+^ ion treatment did not affect cell growth, it significantly inhibited cell motility in a dose-dependent manner (Figure 7B and C). The scratched area was measured, and set at 100% for 0 h. The mean percentage of the total scratched area was calculated. After 18 h of culture, the scratched area was completely closed when the cells were cultured in the absence of Ni^2+^ ion (Figure 7C). However, Ni^2+^ ions significantly inhibited the motility of HSC3 cells and only 45% closure was observed after treatment with 1 mM Ni^2+^ ions. These results indicate that Ni^2+^ ions can inhibit NF-κB activity and thereby reduce the cell motility of OSCC.

## Discussion

Comprehensive analysis of gene expression profiles in OSCC has revealed the constitutive secretion of a number of factors, which have been attributed to aberrant activation of transcription factors in tumor cells [9]. In the present study, we examined the spontaneous secretion of IL-8 in three different OSCC lines. All cell lines secreted IL-8 in a resting state, but with varying levels of secretion. As expected, IL-8 secretion was partially blocked by treatment of cells with specific inhibitors against NF-κB. Extensive study of the transcriptional regulation of IL-8 has revealed that the sequence spanning nucleotides −1 to −133 within the 5′ flanking region of the IL-8 gene is essential [24]. This region contains not only the NF-κB binding site, but also activating protein-1 (AP-1) and CAAT/enhancer-binding protein-binding sites (C/EBP). Consistent with these observations, IL-8 secretion was not completely abrogated by NF-κB specific inhibitors. These results suggest that AP-1 or C/EBP could also contribute to the spontaneous secretion of IL-8.

The initial finding that led to this investigation was that Ni^2+^ ions can bind to the extracellular part of TLR4 and augment the secretion of IL-8 in HUVECs [23]. Several groups reported that the expression of TLR4 and its cognate ligand LPS induces the secretion of soluble factors in oral squamous epithelial cells and OSCC cells [25]. These findings prompted us to examine the effect of Ni^2+^ ions on the secretion of IL-8 in OSCC. The contribution of TLR4 to Ni^2+^ ion-mediated down-regulation of IL-8 secretion was examined by exposure to neutralizing anti-TLR4 antibody and transfection with siRNA. Overall, blockade of the TLR4 pathway had a minor effect on IL-8 secretion, indicating that Ni^2+^ ions exert an inhibitory effect through TLR4-independent mechanisms.

The biological effects of Ni^2+^ ions have been studied in several distinct cell types such as monocytes [26] [27] [28] [29], dendritic cells [30] [31], endothelial cells [28], [32] and epithelial cells [33] [34]. Although most of these studies demonstrated enhanced secretion of soluble factors or expression of cell adhesion molecules, inhibitory effects have also been demonstrated in different experimental settings [26] [27].

NF-κB is the key regulatory factor in adaptive and innate immunity and hence is studied most intensely in relation to Ni^2+^ ions [35] [36]. In contrast to our initial speculation that Ni^2+^ ions could up-regulate the secretion of IL-8 in OSCC, Ni^2+^ ions reduced the secretion of IL-8. Real-time PCR analysis revealed that Ni^2+^ ions exerted an inhibitory effect at the transcriptional level. Moreover, a luciferase assay confirmed the reduction of NF-κB activity by Ni^2+^ ions. To date, the inhibitory effect of Ni^2+^ ions has been observed in monocytes by Lewis et al. [27], who demonstrated that Ni^2+^ ions inhibited LPS-induced p65 binding to DNA in a human monocyte cell line. However, the molecular mechanisms underlying the inhibition of the DNA-binding ability of p65 by Ni^2+^ ions have never been elucidated.

Activation of NF-κB occurs via three different steps: phosphorylation of the inhibitory protein IκB, dimerization, and translocation of NF-κB subunits to the nucleus [11]. Autonomous phosphorylation of p65 has been observed in OSCC and was not altered by addition of Ni^2+^ ions. By contrast, Ni^2+^ ions reduced the nuclear translocation of p50, but not p65, in OSCC cells. These results clearly indicate that NF-κB subunits are regulated independently and that the inhibitory effect of Ni^2+^ ions may be mediated by retrieval of the p50 subunit from the nucleus to the cytoplasm. While p65 possesses a transactivation domain, p50 does not [11]. In this context, the p50/p50 homodimer, but not the p65/p50 heterodimer, inhibits transcriptional activation [11]. At the same time, however, there is substantial evidence that the p50/p50 homodimer also functions as a transcriptional activator. A well-characterized co-activator is B-cell leukemia (Bcl)-3 [37]. Bcl-3 can form a stable complex with the p50/p50 homodimer and stimulate the nuclear import to activate NF-κB target genes. Although Bcl-3 dependent IL-8 regulation is reported in respiratory syncytial virus infected alveolar epithelial cells [38], further study is needed to elucidate the relationship between Ni^2+^ ion-mediated p50 subunit retrieval and reduction of IL-8 secretion.

Based on these observations, we further examined the direct interaction of p50 with Ni^2+^ ions. Surprisingly, the pull-down assay using the Ni^2+^-column successfully precipitated p50. Reduced binding of NF-κB to its target gene has been demonstrated using Ni^2+^ ion-resistant cell lines, [39]; however, to our knowledge, this is the first report demonstrating the interaction of Ni^2+^ ions with the p50 subunit of NF-κB. As the interaction of Ni^2+^ ions and p50 was completely abrogated in the presence of 500 mM of imidazole, we infer that the binding was a His-mediated process. By generating deletion mutants, the interaction domain was revealed to be the N-terminal part of p50. This part of p50 contains 13 His residues, and deletion of the His cluster at positions 108, 110 and 112 totally abrogated the interaction. As deletion of each His residue did not affect the interaction, these residues are thought to contribute to the binding as a cluster (data not shown).

The NF-κB family has five members: p50, p52, p65, c-Rel, and RelB. Of these, p50 and p52 are generated by proteasomal processing from their precursors, p105 and p100, respectively. p105 is a multidomain protein comprising the N-terminal domain (NTD), the dimerization domain (DimD), the ankyrin repeat domain (ARD), and the death domain (DD) (Figure 6A). The 22-amino-acid segment (351–372) immediately adjacent to the DimD in the direction of the C-terminal contains the nuclear localization sequence (NLS). Together the NTD, DimD, and NLS polypeptide constitute the p50 Rel homology domain (RHD) [40]. The His cluster found in this study is localized in the RHD, and Ni^2+^ ion binding could prevent DNA binding and homo- and heterodimerization of p50. To confirm the importance of RHD for Ni^2+^ ion-mediated inhibition, we further established the stable transfectants expressing the WT, N- or C-terminal parts of p50. The inhibitory effect of Ni^2+^ ions was reversed in the N-terminal p50 expressing cells. Western blot analysis of the WT and N-terminal mutant revealed two closely migrating bands. We expected that this could be attributed to differences in phosphorylation status. However, the phosphorylation site of p50 localizes in the C-terminal part of p50 and the nature of these two bands is presently unknown. The precise mechanism of Ni^2+^ ion-mediated NF-κB inhibition needs to be clarified in future studies.

To exert an inhibitory effect by binding directly to p50, Ni^2+^ ions must be incorporated into the cytoplasm. For water-soluble Ni^2+^ compounds, such as NiCl_2_ used in this study, it takes longer for Ni^2+^ ions to accumulate in the nucleus [4]. In our experiments, the inhibitory effect of Ni^2+^ ions was observed shortly after exposure (∼ 1 h) and was expected to be exerted in the cytoplasm. If this is the case, Ni^2+^ ions could bind to p50 in the cytoplasm and prevent nuclear translocation. Although it is known that water-insoluble Ni^2+^ compounds are incorporated by phagocytosis, the method by which water-soluble Ni^2+^ compounds are incorporated is not fully elucidated. In bacterial systems, a receptor for Ni^2+^ ions has been reported [41]. The nickel transporter is composed of five different genes, Nik A to E, which form a periplasmic complex allowing active transport of Ni^2+^ ions into the cytoplasm [41], [42] [43]. By contrast, in mammalian systems, a divalent-cation transporter, a broad substrate range transporter including Ni^2+^ ions, has been identified [44]. The functional importance of this molecule to the Ni^2+^ ion-mediated inhibitory effect needs to be clarified.

The malignancy of tumors can be measured according to metastatic potential and biological behavior. Aberrant activation of NF-κB has been observed in several types of cancer, including OSCC, [12] [45] and is thought to correlate with malignant potential. Loercher et al. compared the gene expression profiles between normal oral epithelium and OSCC by microarray. The results revealed that more than 300 genes were differentially expressed and NF-κB directly or indirectly modulated the gene expression programs relating to proliferation, apoptosis, adhesion and angiogenesis [46]. In fact, suppression of NF-κB activity reduces the growth of tumor cells and inhibits metastasis. He et al. treated the OSCC with NF-κB inhibitor, celastrol. The treatment synergistically augmented the anticancer effect of traditional Chinese medicine, gambogic acid [47]. When the phosphorylation site mutant of IκBα was transfected to murine OSCC, the lack of IκBα degradation resulted in the inactivation of NF-κB and the reduction of cancer cell growth [48]. The prevention of IκBα degradation by proteasomal inhibitor also exerted the growth inhibitory effect on OSCC [49]. Bancroft et al. utilized the epidermal growth factor receptor antagonist to inactivate the NF-κB. Thus treated OSCC reduced the secretion of IL-8, VEGF and led to the growth retardation [50]. Although the mechanisms controlling the incorporation of Ni^2+^ ions are unknown, the fact that Ni^2+^ ions can inhibit NF-κB activity by binding directly to p50 suggests possible clinical applications for Ni^2+^ ions in cancer therapy.
